# Prevalence and Correlates of Exercise Addiction in the Presence vs. Absence of Indicated Eating Disorders

**DOI:** 10.3389/fspor.2020.00084

**Published:** 2020-07-10

**Authors:** Mike Trott, Lin Yang, Sarah E. Jackson, Joseph Firth, Claire Gillvray, Brendon Stubbs, Lee Smith

**Affiliations:** ^1^Cambridge Centre for Sport and Exercise Sciences, Anglia Ruskin University, Cambridge, United Kingdom; ^2^Department of Cancer Epidemiology and Prevention Research, Cancer Control Alberta, Alberta Health Services, Calgary, AB, Canada; ^3^Departments of Oncology and Community Health Sciences, University of Calgary, Calgary, AB, Canada; ^4^Department of Behavioural Science and Health, University College London, London, United Kingdom; ^5^Division of Psychology and Mental Health, University of Manchester, Manchester, United Kingdom; ^6^NICM Health Research Institute, Western Sydney University, Westmead, NSW, Australia; ^7^Centre for Youth Mental Health, University of Melbourne, Melbourne, VIC, Australia; ^8^Cognitive Sports Therapy, Cambridge, United Kingdom; ^9^Cambridge Private Doctors, Nuffield Hospital, Cambridge, United Kingdom; ^10^RC Psych Sports and Exercise Psychiatry Special Interest Group, London, United Kingdom; ^11^Positive Ageing Research Institute (PARI), Anglia Ruskin University, Cambridge, United Kingdom; ^12^Physiotherapy Department, South London and Maudsley NHS Foundation Trust, London, United Kingdom; ^13^Department of Psychological Medicine, Institute of Psychiatry, Psychology and Neuroscience, King's College London, London, United Kingdom

**Keywords:** exercise addiction, exercise dependence, eating disorder, social media, reasons for exercising, exercise, pathological exercise

## Abstract

Despite the many benefits of regular, sustained exercise, there is evidence that exercise can become addictive, to the point where the exerciser experiences negative physiological and psychological symptoms, including withdrawal symptoms upon cessation, training through injury, and the detriment of social relationships. Furthermore, recent evidence suggests that the etiology of exercise addiction is different depending on the presence or absence of eating disorders. The aim of this study was to explore to what extent eating disorder status, body dysmorphic disorder, reasons for exercise, social media use, and fitness instructor status were associated with exercise addiction, and to determine differences according to eating disorder status. The key findings showed that the etiology of exercise addiction differed according to eating disorder status, with variables including social media use, exercise motivation, and ethnicity being uniquely correlated with exercise addiction only in populations with indicated eating disorders. Furthermore, body dysmorphic disorder was highly prevalent in subjects without indicated eating disorders, and could be a primary condition in which exercise addiction is a symptom. It is recommended that clinicians and practitioners working with patients who present with symptoms of exercise addiction should be screened for eating disorders and body dysmorphic disorder before treatments are considered.

## Introduction

Exercise can be defined as “structured, intentional physical activity for improving health and fitness” (Garber et al., 2011). Benefits of regular exercise in adults (18 years and over) include lower risk of all-cause mortality, improved cognitive function, and improvements in several areas of mental health (Ashdown-Franks et al., 2019; Powell et al., 2019).

There is evidence, however, that exercise can become obsessive, compulsive, or addictive, to the point where the exerciser experiences negative physiological and psychological symptoms, including withdrawal symptoms upon cessation, training through injury, and the detriments of social relationships (Symons Downs et al., 2019; Szabo et al., 2019). Several different terms have been used to label this phenomenon, including exercise addiction, exercise dependence, compulsive exercise, and obligatory exercise. For this study, we use the term *exercise addiction* (EA), as it encompasses aspects of both dependence and compulsion (Szabo et al., 2015). Overall prevalence of exercise addiction appears to be 3–14% of the exercising population; however, this varies depending on the population and method of exercise addiction measurement tool (Di Lodovico et al., 2019; Marques et al., 2019; Trott et al., 2020a).

**Figure 1:**
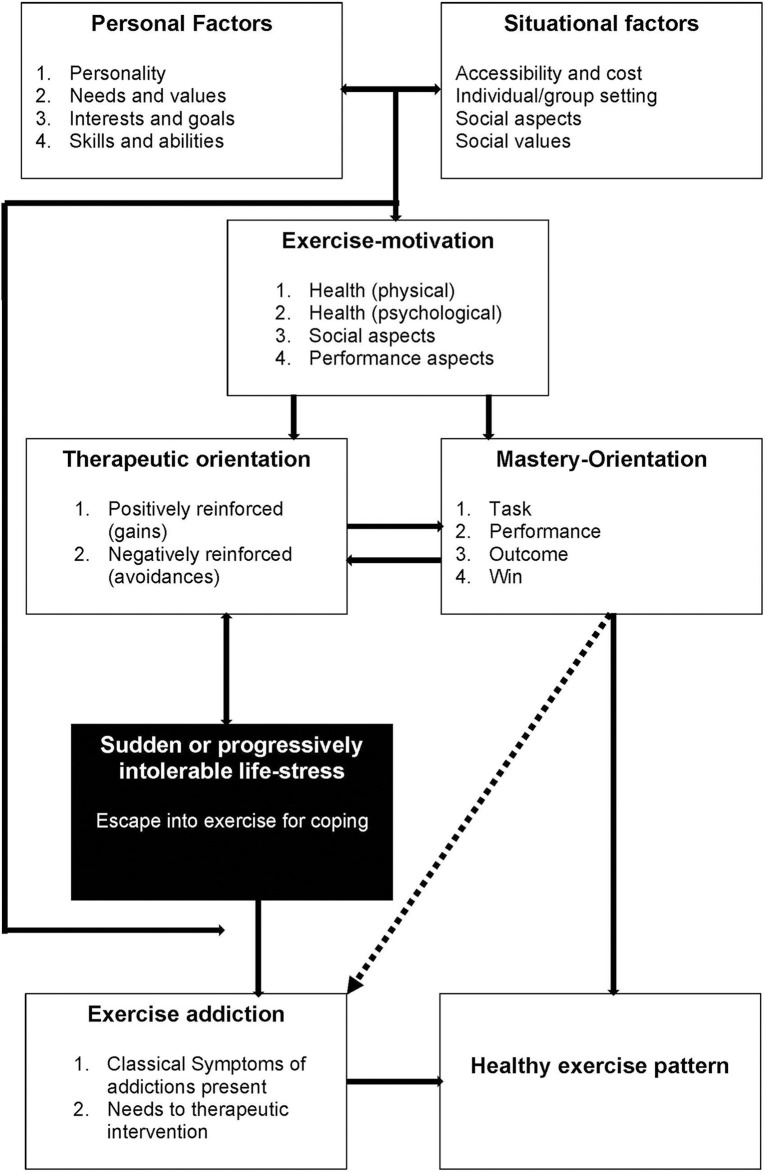
The interactional model of exercise addiction (Egorov and Szabo, 2013).

Many theoretical models have been proposed to explain EA, including the Sympathetic Arousal Hypothesis [(Thompson and Blanton, 1987), the Cognitive Appraisal Hypothesis (Szabo, 1995), the IL-6 model (Hamer and Karageorghis, 2007), Four Phase model (Freimuth et al., 2011), and the Biopsychosocial model (McNamara and McCabe, 2012)]. Most recently, Egorov and Szabo (2013) updated the Cognitive Appraisal Hypothesis with their Interactional Model of EA (Figure 1), which describes a broad range of variables being conducive to developing EA, along with the acknowledgment that several variables' connection may be two-way.

One of the key determinants of EA in the Interactional Model is “sudden or progressively intolerable life-stress.” The most researched of these is the presence (or absence) of eating disorders, with recent evidence concluding that subjects with indicated eating disorders have 3.5x higher risk of developing EA than subjects without indicated eating disorders (Trott et al., 2020b) broadly supporting this model. Further evidence to support this hypothesis, however, is sparse, mainly because the majority of EA literature fails to screen for the presence of eating disorders (Di Lodovico et al., 2019; Marques et al., 2019; Symons Downs et al., 2019). Another condition that could be characterized as an “intolerable life-stress” is the presence of Body Dysmorphic Disorder (BDD), a condition in which a person is concerned about real or perceived physical defects (such as body shape, skin, or hair) as repulsive (Buhlmann et al., 2009; American Psychiatric Association, 2013). Previous studies have shown BDD to be a predictor of exercise addiction in populations without indicated eating disorders (Grandi et al., 2011); however, the strength of this association in populations with indicated eating disorders is unknown. Several other correlates have been shown to associate with BDD, including social media use and sexuality, both of which have been shown to yield more negative body image feelings, with a positive relationship between time spent on social media and negative body feelings (Fardouly and Vartanian, 2016), and heterosexual women and homosexual men demonstrating higher levels of body dissatisfaction (Conner et al., 2004), indicating a potential link between EA, social media use, sexuality, and BDD. These links, however, have not been empirically explored to date.

Another key component of the Interactional Model of EA is “exercise-motivation,” although few studies have explored reasons for exercise in exercise addicted populations. Serier et al. (2018) explored reasons for exercise in subjects with high levels of body dissatisfaction and found that EA subjects scored significantly higher in measures for “exercising for mood” and “enjoyment” compared to non-exercise addicted subjects, broadly supporting the Interactional Model. It has also been suggested that subjects with EA exercise for different reasons depending on the presence or absence of an eating disorder, with subjects with no indicated eating disorders exercising “as an end to itself,” and indicated eating disordered subjects exercising to achieve another goal, such as weight loss (de Coverley Veale, 1987). Evidence to support these differing exercise motivations, however, has not been explored to date.

Further at the beginning of the Interactional Model is “personal” and “situational” factors. Of these, the amount of leisure time physical activity has been consistently shown to positively correlate with exercise addiction risk (Kovacsik et al., 2018). One unique job that could be related to EA is being a fitness instructor (especially group fitness instructors), as they are regularly required to exercise as part of their job, and have been noted at being at higher risk of fitness related injuries, especially when coupled with obligatory exercise tendencies (Thompson et al., 2001); however, whether this directly correlates with increased exercise addiction risk is yet to be explored.

Identifying the extent to which these variables are associated with EA has the potential to support, refute, or suggest modifications to the Interactional Model of EA. Furthermore, identifying how much these associations differ between subjects with and without indicated eating disorders is important, as it allows researchers to understand if there are any differences in the two populations, and therefore have suggested different etiology. The aim of this study, therefore, was to answer the following questions:

1. To what extent is eating disorder status, BDD, reasons for exercise, social media use, and fitness instructor status associated with exercise addiction in line with the Interactional Model?

Based on the Interaction Model, it is hypothesized that eating disorder and BDD status (conditions that could be considered a “sudden or progressively intolerable life-stresses”) have the strongest association with EA. Exercise-motivations are hypothesized to have a smaller association, with the personal and situational factors (fitness instructor status and social media use) showing the smallest associations.

2. Do the associations between these psychological and social variables and exercise addiction differ according to eating disorder status?

We hypothesize that some correlates will differ according to eating disorder status.

Not only will this expand the understanding of exercise addiction, its relationship with eating disorders, and its relationship with the multiple variables described above, it has the potential to inform practitioners working with potentially “at risk” groups, such as physicians and fitness industry workers. Furthermore, this study will either support or refute the most recent model of EA, which will steer the direction of future research.

## Measures and Methods

Study participants were recruited via an international group fitness e-newsletter and through Facebook, Instagram, and Twitter from 8/4/19 to 31/7/19 through social media influencers and through the authors' personal social media accounts. Participants provided informed consent prior to taking part in the survey, including the right to withdraw and access to further support if any of the topics were distressing. To be eligible for the study, participants were required to be adult (>18 years) health club users. Participants were oriented to an online battery of questions hosted through an academic survey website (Jisc Online Surveys, 2020), including measures of age, sex, ethnicity, socio-economic status, life-limiting illness status, exercise addiction, leisure-time physical activity frequency, reasons for exercise, eating disorders, BDD, social media use, body mass index (BMI), and sexuality. Ethical approval was obtained from the Anglia Ruskin University Sport and Exercise Sciences Departmental Ethics Panel (ESPGR-03).

### Participants

Total, 1,864 participants completed the questionnaire. Of these, 199 (10.7%) failed to confirm that they were health club users and were excluded from further analysis. Of the remaining 1,665 participants, the mean age was 35.7 years (*SD* = 10.9), mean self-reported BMI was 23.9 kg/m^2^ (*SD* = 3.9), and 1,428 (85.0%) subjects were female. Full demographic information is shown in Table 1.

**Table 1:** Sample characteristics.

**Variable**	**Total sample^a^**	**Indicated exercise addiction^a^**	**No indicated exercise addiction^a^**
*n*	1,665	511 (30.7%)	1,154 (69.3%)
Age (years)	35.72 (10.92)	34.47 (10.41)	36.28 (11.10)
BMI (kg/m^2^)	23.91 (3.93)	23.64 (4.22)	24.02 (3.79)
Sex (female)	85.00% (*n =* 1,428)	89.4% (*n* = 457)	84.10 (*n =* 971)
EAI^c^ total	21.23 (4.31)	25.91 (1.73)	19.17 (3.40)
Indicated eating disorder (yes)	16.80% (*n* = 279)	32.90% (*n* = 168)	9.60% (*n* = 111)
EAT-26^b^ Total	13.40 (12.43)	20.07 (14.83)	10.45 (9.86)
Fitness instructor (yes)	42.76% (*n* = 712)	42.90% (*n* = 219)	42.70% (*n* = 493)
Exercise hours for leisure (hour/week)	6.46 (4.04)	7.78 (4.50)	5.87 (3.67)
Life limiting illness (yes)	1.14% (*n* = 19)	0.60% (*n* = 3)	1.40% (*n* = 16)
**Sexuality**			
Heterosexual	88.00% (*n* = 1,477)	87.10% (*n* = 445)	89.40% (*n* = 1,032)
Homosexual	4.62% (*n* = 77)	4.50% (*n* = 23)	4.70% (*n* = 54)
Bisexual	4.50% (*n* = 75)	5.70% (*n* = 29)	4.00% (*n* = 46)
Prefer not to say	2.16% (*n* = 36)	2.20 (*n* = 11)	1.40% (*n* = 16)
**Ethnicity**			
White	91.23% (*n* = 1,519)	92.80% (*n* = 474)	90.6% (*n* = 1,045)
Black or African American	0.72% (*n* = 12)	0.40% (*n* = 2)	0.90% (*n* = 10)
Hispanic or Latino	1.62% (*n* = 27)	1.00% (*n* = 5)	1.90% (*n* = 22)
Asian	3.78% (*n* = 63)	3.30% (*n* = 17)	4.00% (*n* = 46)
**Relationship status**			
Single	28.89% (*n* = 481)	34.10% (*n* = 174)	26.60% (*n* = 307)
In a relationship	32.01% (*n* = 533)	31.10% (*n* = 159)	32.40% (*n* = 374)
Married	37.40% (*n* = 630)	33.90% (*n* = 173)	39.60 (*n* = 457)
Widowed	0.24% (*n* = 4)	0.20% (*n* = 1)	0.30% (*n* = 3)
Other	1.02% (*n* = 17)	0.80 (*n* = 4)	0.70% (*n* = 8)
Homeowner status (yes)	57.36% (*n* = 955)	53.40% (*n* = 273)	59.10% (*n* = 682)
BDD^d^ status (indicated)	30.51% (*n* = 508)	48.70% (*n* = 249)	22.40% (*n* = 259)
**REI^e^ subscales**			
Weight control	4.64 (1.27)	5.00 (1.30)	4.48 (1.23)
Fitness	5.88 (0.96)	6.05 (0.94)	5.81 (0.96)
Mood	5.35 (1.36)	5.81 (1.19)	5.14 (1.39)
Health	5.99 (1.02)	6.10 (1.03)	5.94 (1.01)
Attractiveness	4.68 (1.57)	5.13 (1.55)	4.48 (1.54)
Enjoyment	4.55 (1.51)	4.83 (1.52)	4.43 (1.49)
Tone	4.52 (1.51)	4.70 (1.53)	4.44 (1.50)
**SMUIS^f^ subscales**			
Social integration and emotional connection	2.59 (1.12)	2.82 (1.16)	2.49 (1.08)
Integration into social routines	4.11 (1.18)	4.24 (1.20)	4.05 (1.17)

a *Data is presented as mean (standard deviation), unless otherwise stated*.

b *EAI, exercise addiction inventory*.

c *EAT-26, eating attitude test*.

d *BDD, body dysmorphic disorder*.

e *REI, reasons for exercise inventory*.

f *SMUIS, social media use integration scale*.

### Measures

#### Exercise Addiction

The Exercise Addiction Inventory (EAI) (Terry et al., 2004) is a six-item questionnaire that assesses each component of Brown's theory of addiction (Brown, 1993) in an exercise context. Each question is scored on a Likert scale of 1–5, with a higher score indicating higher risk of exercise addiction. Subjects who score ≥24 are classified as “at risk” of exercise addiction (Terry et al., 2004). The EAI has been shown to have good reliability and validity across physically active populations (Terry et al., 2004; Griffiths et al., 2015; Lichtenstein and Jensen, 2016) and shows good internal reliability in the current study (α = 0.74).

Note: Despite having a cut-off score, the EAI was used as a continuous variable indicting severity of exercise addiction risk because there are no clinically recognized diagnostic criteria for exercise addiction (American Psychiatric Association, 2013).

#### Social Media Use

Social media use was measured using the Social Media Use Integration Scale (SMUIS) (Jenkins-Guarnieri et al., 2013), a 10-item questionnaire with two sub-scales: social integration and emotional connection and integration into social routines. Each question is scored on a Likert scale of 1–6, with higher scores in each sub-scale indicating higher levels of its respective sub-scale. The SMUIS has shown good validity across several age ranges (Jenkins-Guarnieri et al., 2013; Maree, 2017) and shows excellent internal consistency in the current study (social integration and emotional connection sub-scale Cronbach's α = 0.88; integration into social routines sub-scale Cronbach's α = 0.81).

#### Reasons for Exercise

Reasons for exercise was measured using the Reasons for Exercise Inventory (REI) (Silberstein et al., 1988), a 24-item questionnaire with seven sub-scales: weight control, fitness, mood, health, attractiveness, enjoyment, and tone. Each question is scored on a Likert scale of 1–7, with higher scores in each sub-scale indicating higher levels in the respective sub-scale. The REI has been validated across several populations (Silberstein et al., 1988; Cash et al., 1994) and in the current study shows good internal consistency (Cronbach's αs: weight control α = 0.61; fitness α = 0.83; mood α = 0.86; health α = 0.86; attractiveness α = 0.85; enjoyment α = 0.82; tone α = 0.79).

#### BDD

BDD was measured using the Body Dysmorphic Disorder Questionnaire (BDDQ) (Phillips, 2005), a questionnaire based on the *DSM-IV* (American Psychiatric Association, 2000) diagnostic criteria for BDD. Classification of BDD is made based on answering positively to questions 1 and 2, at least one part of question 3, and indicating spending one or more hours each day thinking about their appearance. The questionnaire has excellent reported sensitivity (94%) and specificity (90%) in non-clinical community populations (Brohede et al., 2013).

#### Eating Disorder Symptoms

Eating disorder symptomology was measured using the Eating Attitudes Test 26 (EAT-26) (Garner et al., 1982), a 26-item questionnaire scored on a Likert scale of 1–6. A score of ≥20 is sufficient to be classified as having possible pathological eating behaviors. The EAT-26 has been well-validated in athletic populations (Doninger et al., 2005; Pope et al., 2015) and has shown excellent internal consistency in the current study (Cronbach's α = 0.91).

#### Health Club User

Participants were required to answer yes/no to indicate whether they were a current health club user.

#### Fitness Instructor

Participants were required to answer yes/no to indicate if they were currently a fitness instructor.

#### Leisure-Time Physical Activity

Participants were required to indicate how many hours per week they participated in physical activity (if the subject was a fitness instructor, this did not include exercise hours as part of work).

### Data Analysis

All data were analyzed using SPSS Version 26 (IBM Corp., 2019).

Exercise addiction prevalence was also calculated in all the total sample and both indicated and non-indicated eating disorder populations.

A hierarchical multiple linear regression was run on the total sample to determine if the addition of variables significantly added to the total model with EAI score (as a continuous variable) as the dependent variable. The variables were added to the previous models in the following order:

Model 1: Age, gender, BMI, ethnicity, life limiting illness
Model 2: Eating disorder status
Model 3: BDD status
Model 4: Reasons for exercise (all items)
Model 5: Fitness instructor status
Model 6: Social media use (all items)
Model 7: Sexuality
Model 8: Exercise hours for leisure
Model 9: Relationship status

Furthermore, a linear regression was used to analyse associations between exercise addiction score (as a continuous variable) and: age, sex, BMI, ethnicity, eating disorder status, homeowner status, relationship status, both subscales of the SMUIS, all subscales of the REI, being a fitness instructor, leisure time physical activity, sexuality, and BDD status in two populations:

1. Indicated eating disorders (defined as scoring ≥20 in the EAT-26)
2. No indicated eating disorders (defined as scoring <20 in the EAT-26)

Any missing data was tested for randomness via Little's MCAR test (Little, 1988), and if confirmed random, deleted listwise from all regression analyses.

In order to explore whether associations varied according to eating disorder status, we repeated the multivariable analysis (model 9) in a series of linear regression models adding the interaction term (eating disorder status*respective variable) between eating disorder status and each potential correlate in turn (e.g., in the first analysis we included all variables in model 9 with the addiction of the variable “eating disorder status*age”; in the second analysis we included all variables in model 9 with the addiction of the variable “eating disorder status*gender”, etc.).

## Results

### Exercise Addiction Prevalence

The prevalence of exercise addiction, as defined by a score of ≥24 on the EAI (Terry et al., 2004), in the total sample was 30.7% (95%CI = 28.5–33.0%), 60.2% (95%CI = 54.2–66.0%) in the population who had an indicated eating disorders, and 24.7% (95%CI = 22.5–27.1%) in the population who had no indicated eating disorders.

### Regression Assumption Testing

There was linearity in all samples as assessed by partial regression plots and a plot of studentized residuals against the predicted values. There was independence of residuals in all populations, as assessed by a Durbin-Watson statistic of 2.108, 1.087, and 2.036 in the total sample, indicated ED and no indicated ED samples, respectively. Homoscedasticity was as assessed by visual inspection of a plot of studentized residuals vs. unstandardized predicted values, with evidence of homoscedasticity in all three samples. There was no evidence of multicollinearity in any sample, as assessed by tolerance values >0.1. There were 23 studentized deleted residuals >±3 standard deviations, which were kept in the analysis. The assumption of normality was met, as assessed by a Q-Q Plot. The Little's MCAR test confirmed that all missing data was random (*p* = 0.07), and therefore were listwise deleted from all regression analyses.

### Hierarchical Multiple Regression

In the total sample, each model significantly added to the total R^2^, apart from models 5, 7, and 9 (the respective addition of fitness instructor status, sexuality, and relationship status into the previous model). The final multiple regression model (model 9) was statistically significant [*F*_(29, 1, 500)_ = 16.227, *p* ≤ 0.001, adj. *R*^2^ = 0.224]. The variables BMI, life limiting illness, being a fitness instructor, exercise hours for leisure, eating disorder status, REI “mood” and “enjoyment” subscales, SMUIS social integration and emotional connection subscale, BDD status, ethnicity black and Asian (vs. white) added significantly to the prediction (*p* ≤ 0.05). Full coefficient results and changes in *R*^2^ are shown in Table 2.

**Table 2:** Hierarchical regression in the total sample (exercise addiction inventory scores as the dependent variable).

	**Model 1**		**Model 2**		**Model 3**		**Model 4**		**Model 5**		**Model 6**		**Model 7**		**Model 8**		**Model 9**	
	***R*^**2**^**	***R*^**2**^ change**	***R*^**2**^**	***R*^**2**^ change**	***R*^**2**^**	***R*^**2**^ change**	***R*^**2**^**	***R*^**2**^ change**	***R*^**2**^**	***R*^**2**^ change**	***R*^**2**^**	***R*^**2**^ change**	***R*^**2**^**	***R*^**2**^ change**	***R*^**2**^**	***R*^**2**^ change**	***R*^**2**^**	***R*^**2**^ change**
	**0.027**	**NA**	**0.079**	0.052**	**0.098**	0.019**	**0.180**	0.082**	**0.180**	**0.000**	**0.184**	0.004**	**0.184**	**0.000**	**0.226**	0.042**	**0.224**	**−0.002**
Variable	Beta coefficients (95%CI)	*p*	Beta coefficients (95%CI)	*p*	Beta coefficients (95%CI)	*p*	Beta coefficients (95%CI)	*p*	Beta coefficients (95%CI)	*p*	Beta coefficients (95%CI)	*p*	Beta coefficients (95%CI)	*p*	Beta coefficients (95%CI)	*p*	Beta coefficients (95%CI)	*p*
Age	−0.106 (−0.156; −0.056)	<0.001**	−0.084 (−0.133; −0.036)	0.001	−0.056 (−0.105; −0.006)	0.027	−0.044 (−0.093; 0.005)	0.081	−0.045 (−0.094; 0.004)	0.073	−0.038 (−0.087; 0.012)	0.138	−0.036 (−0.086; 0.014)	0.156	−0.046 (−0.095; 0.002)	0.061	−0.042 (−0.102; 0.017)	0.165
Sex	−0.052 (−0.103; −0.002)	<0.001**	−0.022 (−0.071; 0.027)	0.385	−0.005 (−0.054; 0.044)	0.843	0.020 (−0.029; 0.069)	0.417	0.020 (−0.029; 0.068)	0.432	0.021 (−0.028; 0.070)	0.404	0.023 (−0.030; 0.075)	0.392	0.004 (−0.047; 0.055)	0.881	0.004 (−0.048; 0.055)	0.888
BMI	−0.064 (−0.115; −0.014)	0.012	−0.055 (−0.104; −0.006)	0.027	−0.067 (−0.115; −0.018)	0.007	−0.071 (−0.118; −0.025)	0.003	−0.071 (−0.118; −0.025)	0.003	−0.074 (−0.121; −0.028)	0.002	−0.074 (−0.121; −0.028)	0.002	−0.049 (−0.094; −0.003)	0.037	−0.048 (−0.094; −0.002)	0.039
Ethnicity: white vs. Hispanic	−0.013 (−0.062; 0.037)	0.613	−0.011 (−0.059; 0.037)	0.659	−0.011 (−0.059; 0.036)	0.643	−0.016 (−0.061; 0.030)	0.494	−0.017 (−0.063; 0.0.029)	0.465	−0.013 (−0.058; 0.033)	0.585	−0.016 (−0.062; 0.030)	0.500	−0.011 (−0.056; 0.033)	0.614	−0.011 (−0.056; 0.033)	0.620
Ethnicity: white vs. black	−0.091 (−0.140; −0.041)	<0.001**	−0.099 (−0.147; −0.051)	<0.001**	−0.094 (−0.142; −0.046)	<0.001**	−0.066 (−0.112; −0.020)	0.005	−0.066 (−0.112; −0.020)	0.005	−0.065 (−0.111; −0.019)	0.006	−0.068 (−0.113; −0.022)	0.004	−0.071 (−0.115; −0.026)	0.002	−0.071 (−0.116; −0.027)	0.002
Ethnicity: white vs. Asian	−0.020 (−0.070; 0.029)	0.423	−0.021 (−0.070; 0.027)	0.388	−0.015 (−0.063; 0.033)	0.530	−0.026 (−0.072; 0.020)	0.270	−0.025 (−0.071; 0.021)	0.290	−0.027 (−0.073; 0.019)	0.253	−0.029 (−0.075; 0.017)	0.216	−0.045 (−0.090; 0.000)	0.050	−0.048 (−0.094; −0.003)	0.038
Ethnicity: white vs. “other”	0.001 (−0.049; 0.051)	0.970	0.005 (−0.043; 0.053)	0.842	0.009 (−0.039; 0.057)	0.708	−0.005 (−0.050; 0.041)	0.843	−0.004 (−0.050; 0.042)	0.855	−0.004 (−0.050; 0.041)	0.855	−0.004 (−0.050; 0.041)	0.850	−0.005 (−0.050; 0.039)	0.817	−0.006 (−0.051; 0.039)	0.795
Life limiting illness	−0.040 (−0.089; 0.010)	0.120	−0.046 (−0.094; 0.003)	0.065	−0.041 (−0.089; 0.007)	0.096	−0.046 (−0.092; 0.000)	0.048	−0.046 (−0.092; 0.000)	0.050	−0.048 (−0.094; −0.002)	0.041	−0.051 (−0.097; −0.005)	0.031	−0.055 (−0.100; −0.011)	0.015	−0.055 (−0.100; −0.010)	0.017
Eating disorder status			0.233 (0.185; 0.282)	<0.001**	0.163 (0.109; 0.217)	<0.001**	0.135 (0.082; 0.188)	<0.001**	0.136 (0.083; 0.189)	<0.001**	0.135 (0.083; 0.188)	<0.001**	0.134 (0.081; 0.187)	<0.001**	0.106 (0.054; 0.158)	<0.001**	0.106 (0.054; 0.159)	<0.001**
BDD status					0.162 (0.107; 0.218)	<0.001**	0.123 (0.069; 0.178)	<0.001**	0.123 (0.068; 0.178)	<0.001**	0.117 (0.062; 0.172)	<0.001**	0.119 (0.064; 0.174)	<0.001**	0.112 (0.058; 0.165)	<0.001**	0.111 (0.057; 0.164)	<0.001**
REI weight control							0.067 (0.012; 0.122)	0.018	0.067 (0.012; 0.122)	0.018	0.065 (0.010; 0.120)	0.020	0.064 (0.009; 0.119)	0.023	0.060 (0.006; 0.113)	0.030	0.060 (0.006; 0.114)	0.030
REI fitness							0.067 (0.007; 0.127)	0.028	0.065 (0.005; 0.125)	0.035	0.062 (0.002; 0.122)	0.043	0.060 (0.000; 0.120)	0.052	0.043 (−0.016; 0.102)	0.154	0.044 (−0.015; 0.103)	0.144
REI mood							0.205 (0.150; 0.260)	<0.001**	0.205 (0.150; 0.260)	<0.001**	0.202 (0.147; 0.257)	<0.001**	0.201 (0.146; 0.256)	<0.001**	0.200 (0.147; 0.254)	<0.001**	0.199 (0.146; 0.253)	<0.001**
REI health							−0.051 (−0.115; 0.014)	0.122	−0.050 (−0.115; 0.014)	0.125	−0.036 (−0.101; 0.029)	0.281	−0.035 (−0.101; 0.030)	0.288	−0.021 (−0.084; 0.043)	0.521	−0.021 (−0.085; 0.043)	0.523
REI attractiveness							0.048 (−0.008; 0.104)	0.096	0.050 (−0.007; 0.106)	0.084	0.034 (−0.023; 0.091)	0.236	0.038 (−0.019; 0.095)	0.195	0.049 (−0.007; 0.105)	0.084	0.049 (−0.007; 0.106)	0.085
REI enjoyment							0.105 (0.054; 0.156)	<0.001**	0.101 (0.049; 0.152)	<0.001**	0.094 (0.042; 0.146)	<0.001**	0.095 (0.043; 0.146)	<0.001**	0.070 (0.019; 0.121)	0.007	0.068 (0.017; 0.119)	0.009
REI tone							−0.038 (−0.086; 0.010)	0.121	−0.040 (−0.088; 0.008)	0.105	−0.040 (−0.088; 0.008)	0.099	−0.041 (−0.089; 0.007)	0.092	−0.044 (−0.091; 0.002)	0.063	−0.044 (−0.091; 0.003)	0.068
Fitness instructor status									0.024 (−0.023; 0.071)	0.323	0.018 (−0.029; 0.065)	0.460	0.017 (−0.030; 0.064)	0.485	0.063 (0.016; 0.110)	0.009	0.063 (0.016; 0.111)	0.009
SMUIS social integration and emotional connection											0.086 (0.024; 0.148)	0.006	0.085 (0.023; 0.148)	0.007	0.084 (0.024; 0.145)	0.006	0.083 (0.023; 0.144)	0.007
SMUIS integration into social routines											−0.024 (−0.084; 0.036)	0.430	−0.024 (−0.084; 0.036)	0.436	−0.004 (−0.063; 0.065)	0.884	−0.003 (−0.061; 0.056)	0.932
Sexuality: heterosexual vs. homosexual													0.013 (−0.062; 0.087)	0.739	−0.013 (−0.086; 0.059)	0.723	−0.013 (−0.086; 0.061)	0.735
Sexuality: heterosexual vs. bisexual													0.024 (−0.042; 0.089)	0.481	0.001 (−0.063; 0.065)	0.983	0.002 (−0.062; 0.066)	0.950
Sexuality: heterosexual vs. “prefer not to say”													0.045 (−0.010; 0.099)	0.106	0.032 (−0.021; 0.085)	0.242	0.031 (−0.022; 0.085)	0.248
Exercise hours for leisure (hour/week)															0.217 (0.170; 0.264)	<0.001**	0.214 (0.167; 0.262)	<0.001**
Relationship status: single vs. “in a relationship”																	0.001 (−0.075; 0.035)	0.969
Relationship status: single vs. married																	−0.020 (−0.088; 0.038)	0.477
Relationship status: single vs. widowed																	−0.025 (−0.033; 0.058)	0.442
Relationship status: single vs. “other”																	0.013 (−0.033; 0.058)	0.586

** *Indicates significant to less than 0.01*.

### Indicated vs. No-Indicated Eating Disorders Sub-groups Multiple Regression

Both populations' full regression models were statically significant [indicated eating disorders = *F*_(27, 231)_ = 2.995, *p* ≤ 0.001, adj. *R*^2^ = 0.173; no indicated eating disorders = *F*_(28, 1, 242)_ = 12.383, *p* ≤ 0.001, adj. *R*^2^ = 0.201]. In the indicated eating disorders population, the variables BMI, SMUIS social integration, and emotional connection subscale, and ethnicity black and Asian (vs. white) added significantly to the regression model (*p* ≤ 0.05). In the no indicated eating disorders population, the variables REI “mood” and “enjoyment” subscales, being a fitness instructor, exercise hours per week, and BDD status added significantly to the regression model (*p* ≤ 0.05). Full coefficients for both populations are shown in Table 3.

**Table 3:** Multiple linear regression summary of independent variables (dependent variable = exercise addiction inventory total score).

	**Indicated eating disorders**		**No-indicated eating disorders**	
	**Beta coefficients (95%CI)**	***p*-value**	**Beta coefficients (95%CI)**	***p*-value**
Age	0.027 (−0.140; 0.194)	0.751	−0.046 (−0.112; 0.020)	0.171
Sex	0.059 (−0.067; 0.184)	0.357	−0.011 (−0.069; 0.047)	0.708
BMI^a^	−0.189 (−0.316; −0.062)	0.004**	−0.013 (−0.064; 0.038)	0.616
Life limiting illness	−0.131 (−0.254; −0.008)	0.038*	−0.041 (−0.091; 0.009)	0.107
Fitness instructor status	−0.068 (−0.176; 0.060)	0.297	0.093 (0.040; 0.146)	0.001**
Exercise hours for leisure	0.156 (0.031; 0.280)	0.014*	0.246 (0.194; 0.298)	<0.001**
Homeowner status	0.009 (−0.138; 0.156)	0.903	0.006 (−0.056; 0.068)	0.852
REI weight control	0.008 (−0.125; 0.140)	0.907	0.065 (0.007; 0.122)	0.028*
REI fitness	0.100 (−0.081; 0.282)	0.277	0.024 (−0.039; 0.087)	0.456
REI mood^a^	0.055 (−0.102; 0.213)	0.491	0.244 (0.185; 0.302)	<0.001**
REI health	0.022 (−0.182; 0.226)	0.833	−0.015 (−0.082; 0.053)	0.668
REI attractiveness^a^	−0.046 (−0.188; 0.097)	0.528	0.078 (0.017; 0.139)	0.013*
REI enjoyment	0.028 (−0.117; 0.174)	0.700	0.075 (0.019; 0.131)	0.009*
REI tone	0.089 (−0.032; 0.211)	0.149	−0.078 (−0.130; −0.026)	0.003**
SMUIS social integration and emotional connection	0.204 (0.048; 0.361)	0.011*	0.067 (−0.001; 0.135)	0.054
SMUIS integration into social routines	−0.124 (−0.282; 0.033)	0.121	0.022 (−0.043; 0.088)	0.509
BDD status	0.056 (−0.076; 0.187)	0.405	0.103 (0.050; 0.157)	<0.001*
Sexuality: Heterosexual vs. homosexual	0.038 (−0.221; 0.297)	0.773	−0.025 (−0.104; 0.054)	0.539
Sexuality: heterosexual vs. bisexual	0.085 (−0.149; 0.319)	0.476	−0.029 (−0.098; 0.040)	0.411
Sexuality: heterosexual vs. “prefer not to say”	0.135 (−0.037; 0.308)	0.123	0.016 (−0.042; 0.074)	0.595
Relationship status: single vs. “in a relationship”	−0.051 (−0.193; 0.090)	0.476	−0.005 (−0.066; 0.057)	0.884
Relationship status: single vs. married	−0.070 (−0.227; 0.087)	0.381	−0.013 (−0.084; 0.058)	0.724
Relationship status: single vs. widowed	NA	NA	0.015 (−0.036; 0.065)	0.567
Relationship status: single vs. “other”	−0.026 (−0.147; 0.096)	0.675	−0.006 (−0.056; 0.045)	0.827
Ethnicity: white vs. Hispanic	−0.118 (−0.234; 0.003)	0.045*	0.004 (−0.046; 0.054)	0.871
Ethnicity: white vs. black^a^	−0.320 (−0.443; −0.196)	<0.001**	−0.005 (−0.055; 0.044)	0.832
Ethnicity: white vs. Asian	−0.139 (−0.261; −0.017)	0.026*	−0.024 (−0.075; 0.027)	0.358
Ethnicity: white vs. “other”	−0.038 (−0.156; 0.080)	0.524	−0.001 (−0.049; 0.051)	0.959

* *P < 0.05*;

** *P < 0.01*.

a *Interaction terms showed correlate differs by eating disorder status*.

### Eating Disorder Interaction Effects

There were significant interactions between eating disorder status and BMI, exercising for mood, exercising for attractiveness, and ethnicity (black vs. white). Full interaction data are shown in Table 4.

**Table 4:** Interaction effects between independent variables and eating disorder status (dependent variable = exercise addiction inventory total score).

**Independent variable by eating disorder status (indicated/not indicated)**	**Beta coefficients (95%CI)**	***p*-value**
Age	0.001 (−0.051; 0.052)	0.993
Sex^a^	0.017 (−0.030; 0.064)	0.480
BMI	−0.260 (−0.497; −0.023)	0.032
Life limiting illness^b^	−0.025 (−0.076; 0.025)	0.331
Fitness instructor status^c^	−0.053 (−0.112; 0.006)	0.081
Exercise hours for leisure	−0.069 (−0.162; 0.023)	0.140
Homeowner status^d^	−0.022 (−0.885; 0.045)	0.516
REI weight control	−0.185 (−0.403; 0.034)	0.097
REI fitness	−0.057 (−0.293; 0.179)	0.637
REI mood	−0.314 (−0.510; −0.119)	0.002**
REI health	−0.148 (−0.369; 0.073)	0.190
REI attractiveness	−0.196 (−0.365; −0.027)	0.023*
REI enjoyment	−0.089 (−0.217; 0.039)	0.172
REI tone	0.094 (−0.055; 0.243)	0.217
SMUIS social integration and emotional connection	−0.007 (−0.128; 0.114)	0.911
SMUIS integration into social routines	−0.113 (−0.281; 0.055)	0.187
BDD status^*e*^	−0.032 (−0.130; 0.066)	0.521
Sexuality: heterosexual vs. homosexual^f^	−0.099 (−0.246; 0.048)	0.187
Sexuality: heterosexual vs. bisexual^g^	0.041 (−0.010; 0.092)	0.112
Sexuality: heterosexual vs. “prefer not the say”^h^	0.021 (−0.029; 0.071)	0.413
Relationship status: single vs. “in a relationship”^i^	0.004 (−0.060; 0.068)	0.902
Relationship status: single vs. married^j^	−0.013 (−0.068; 0.042)	0.645
Relationship status: single vs. widowed^k^	NA (not enough data)	–
Relationship status: single vs. “other”^l^	−0.002 (−0.068; 0.064)	0.953
Ethnicity: white vs. Hispanic^m^	−0.043 (−0.091; 0.005)	0.077
Ethnicity: white vs. black^n^	−0.104 (−0.159; −0.049)	<0.001**
Ethnicity: white vs. Asian^o^	−0.048 (−0.098; 0.002)	0.059
Ethnicity: white vs. “other”^p^	−0.019 (−0.067; 0.029)	0.442

* *P < 0.05*;

** *P < 0.01; Dichotomous variable coding*:

a *Female = 0, Male = 1*.

b *Life limiting illness: No = 0, Yes = 1*.

c *Fitness instructor: No = 0, Yes = 1*.

d *Homeowner status: No = 0, Yes = 1*.

e *BDD status: No = 0, Yes = 1*.

f *Sexuality: Heterosexual = 0, Homosexual = 1*.

g *Sexuality: Heterosexual = 0, Bisexual = 1*.

h *Sexuality: Heterosexual = 0, “prefer not to say” = 1*.

i *Relationship status: Single = 0, in a relationship = 1*.

j *Relationship status: Single = 0, married = 1*.

k *Relationship status: Single = 0, widowed = 1*.

l *Relationship status: Single = 0, other = 1*.

m *Ethnicity: White = 0, Hispanic = 1*.

n *Ethnicity: White = 0, black = 1*.

o *Ethnicity: White = 0, Asian = 1*.

p *Ethnicity: White = 0, other= 1*.

## Discussion

The present study explored the prevalence of exercise addiction among fitness club users, the extent to which age, BMI, gender, sexuality, social media use, BDD, fitness instructor status, eating disorder status, and reasons for exercise were associated with exercise addiction scores, and whether these correlates differed according to eating disorder status. The prevalence of exercise addiction in the total sample was 30.7%, with prevalence rates differing largely according to eating disorder status (indicated eating disorders 60.2%; no indicated eating disorders 24.7%). Characteristics associated with higher exercise addiction scores in multivariable models included: indicated eating disorder, being a fitness instructor, leisure-time physical activity, exercising to improve mood, enjoyment, and for weight control, indicated BDD, and using social media for social integration and emotional connection. Characteristics associated with lower exercise addiction scores included: a higher BMI, reporting a life-limiting illness, and ethnicity (black vs. white and Asian vs. white). There were significant interactions between eating disorder status and BMI, exercising for mood, exercising for attractiveness, and ethnicity (black vs. white).

### Total Sample

The hierarchical regression showed that the addition of all variables into the model significantly increased the *R*^2^, apart from the addition of fitness instructor status, sexuality, and relationship status, indicting their limited significance in explaining the total variance in EAI scores.

As hypothesized, the strength of associations of the two variables that could be interpreted as “sudden or progressively intolerable life-stress” (eating disorder status and BDD status) in the Interactional Model of EA were among the strongest. This concurs with several studies that have shown that eating disordered subjects suffer from higher EA (Fietz et al., 2014; Trott et al., 2020b), and several studies that show that negative self-body image is positively correlated with exercise addiction (Klein et al., 2004; Ertl et al., 2018). Moreover, this provides initial evidence that these two conditions could be listed in the Interactional Model as possible intolerable life-events. Another variable that had one of the strongest associations with EA was exerting to modify mood. Although this could be interpreted as “psychological health” on the Interactional Model, it also could be dealing with a sudden or progressively intolerable life stress, such as depression or anxiety, which would place this variable into this part of the model. Furthermore, this association broadly concurs with previous studies that have found that exercising for mood is positively correlated with exercise addiction (Serier et al., 2018). Due to this, we propose a modification to the Interactional Model: adding a direct link between “exercise motivation” and “sudden or progressive intolerable life-stress.”

Unsurprisingly, leisure-time physical activity was a significant correlate of higher scores of exercise addiction, which concurs with the literature (Hausenblas and Downs, 2002; Adams et al., 2003; Allegre et al., 2007; Costa et al., 2013). One possible mechanism of this relationship could be the desire to increase levels of β-endorphins through increasing amounts of exercise, leading to a relative feeling of euphoria post-exercise (Leuenberger, 2006). Studies in other addictions have suggested that the endogenous opioid system is a key factor in generating addictions (O'Brien, 2004).

### Analysis According to Eating Disorder Status

Lower BMI, using social media for social integration and emotional connection, and ethnicity (white vs. black, Hispanic, and Asian) were only positively associated with higher exercise addiction scores among health club users with indicated eating disorders, and fitness instructor status, exercising to improve mood, attractiveness, exercising for enjoyment, and BDD status were only associated with higher exercise addiction scores among health club users without an indicated eating disorder.

Lower BMI was a correlate of higher exercise addiction scores only in health club users who had an indicated eating disorder. This is consistent with the eating disorder literature which states that striving for a lower body weight (and therefore a lower BMI) via excessive exercise is a common symptom of both anorexia and bulimia nervosa (Abraham, 2016), adding to the evidence that exercise levels should be closely monitored in subjects with indicated eating disorders.

Participants who identified as fitness instructors had a slightly higher risk of higher exercise addiction scores than health club users who did not identify as fitness instructors; however, in the sub-populations this was only present in participants who showed no indicated eating disorders. One possible reason is because of the expectation of fitness instructors to exercise as part of their role, and the expectation of superior levels of fitness compared to regular health club users (Thompson et al., 2001); more research is needed to test this hypothesis. A recent study reported that fitness instructors are frequently worried about members in their centers who exhibit EA tendencies but are unsure on how to deal with these people (Colledge et al., 2020). These results suggest that fitness instructors should monitor their peers as well as their members.

Participants who reported exercising to improve their mood, to be more attractive, weight control, tone, and for enjoyment had higher exercise addiction scores; however, this was only seen in participants who had no indicated eating disorders. This is broadly consistent with previous studies that have found that exercising for mood, appearance, and enjoyment is positively correlated with exercise addiction (Serier et al., 2018). The finding that exercising for these reasons was only significant in participants without an indicated eating disorder was interesting as previous studies have found that people who exercise for mood and appearance reasons are more likely to demonstrate eating pathology (Macfarlane et al., 2016). This adds initial evidence that the links between exercise motivation and EA are different according to eating disorder status, and therefore indicates differing etiology for EA for the two sub-populations. This is important as if the two sub-populations have differing EA etiologies, then it is possible that therapeutic interventions would need to be different. Further research exploring potential mediating relationships between reasons for exercise, eating disorders, and exercise addiction would greatly add to the knowledge in this area.

Participants with indicated BDD were significantly more likely to yield higher exercise addiction scores, but only in participants without indicated eating disorders. Although this concurs with several studies that have shown that negative self-body image is positively correlated with exercise addiction (Klein et al., 2004; Ertl et al., 2018), this is the first study to our knowledge to show that this is not the case in populations with indicated eating disorders. This suggests that BDD is a primary condition in which exercise addiction is a symptom. This is important, as if BDD is a primary condition where EA is a symptom, then the treatment of BDD should yield lower levels of EA. It is therefore recommended that patients presenting with EA symptoms (who do not show evidence of eating disorders) should be screened for BDD before any treatments can be considered.

In the group with indicated eating disorders, participants from ethnic minorities (black, Hispanic, and Asian vs. being white) yielded higher exercise addiction scores. This is the first time such a finding has been reported, and this could be because of the long-recognized limited treatment barriers to eating disorders that subjects from ethnic minorities face (Cachelin et al., 2001; Becker et al., 2003; Coffino et al., 2019). Confirmatory and causal exploration is needed to confirm this relationship and explore interventions to address this.

### Exercise Addiction Prevalence

The prevalence of exercise addiction was high in this sample, with 30.7% being classified as at risk of exercise addiction. Prevalence rates differed largely according to eating disorder status, with participants with indicated eating disorders yielding more than double the prevalence rates than those with no indicated eating disorders. These results are broadly in agreement with a recent meta-analysis that showed subjects with indicated eating disorders are over 3.5 times more likely to also have exercise addiction (Trott et al., 2020b). The overall exercise addiction prevalence rate is higher than in several reviews that have estimated prevalence between 3 and 14% (Di Lodovico et al., 2019; Marques et al., 2019). One potential reason could be because of the recruitment strategy and specific population group; this study used social media as a means of recruitment and was restricted to health club users, which is unique in this area of research. This is supported by our finding that using social media for social integration and emotional connection was a significant predictor for higher exercise addiction scores. Social media use has been shown to elicit feelings of negative body image (Perloff, 2014; Fardouly and Vartanian, 2016), which has been shown to be associated with exercise addiction. Social media is an appropriate platform to recruit from, however, primarily due to the number of people who routinely engage in social media. Recent data suggests that 2.2 billion people use social media on a daily basis (Facebook, 2019). The role of social media's influence in the etiology of exercise addiction warrants further exploration.

### Limitations and Strengths

This study had several limitations. Firstly, due to the cross-sectional nature of the study design, the direction of correlation (and therefore causality) is impossible to determine. Further longitudinal analysis is required to determine the direction of the observed correlations. Secondly, it has been reported that the EAI can yield false-positive results in elite athletes (Szabo et al., 2015), and it is unknown whether the EAI over-estimates exercise addiction prevalence in other highly active populations who exercise as part of their job, such as fitness instructors. Further validation of this questionnaire in this sub-population is warranted. Thirdly, the variables accounted for a low percentage of the total variation. Moreover, the sample was restricted to health club users who were recruited via social media, making the generalization of the findings across populations difficult. Despite these limitations, the large sample size, novelty of measured correlates, and our findings that significant variables of EA vary according to eating disorder status mean that this study adds significant knowledge to the current EA literature.

## Conclusion

The key findings from this study suggest a direct link between exercise motivations and EA, especially if the reason for exercising is to modify mood state. It is suggested that exercising to modify mood state, eating disorder status, and BDD status be included in the intolerable life-stress section of the Interactional Model of EA.

Furthermore, this study shows that the etiology of EA differs according to eating disorder status, with variables including social media use, exercise motivation, and ethnicity being uniquely correlated with EA only in populations with indicated eating disorders. Furthermore, BDD is also highly prevalent in subjects without indicated eating disorders and could be a primary condition in which exercise addiction is a symptom. It is recommended that clinicians and practitioners working with patients who present with symptoms of EA should be screened for eating disorders and BDD before treatments are considered, as both eating disorders and BDD have considerably higher co-morbid outcomes than EA, and therefore need to be treated as a primary condition. Furthermore, treatment programs already exist for these two primary conditions and therefore can be implemented easier. The development of screening tools that are able to stratify these populations would be beneficial to both researchers and practitioners.

## Data Availability Statement

The datasets generated for this study are available on request to the corresponding author.

## Ethics Statement

The studies involving human participants were reviewed and approved by Anglia Ruskin University Sport and Exercise Sciences Departmental Ethics Panel (ESPGR-03). The patients/participants provided their written informed consent to participate in this study.

## Author Contributions

MT and LS: study design, data collection, data analysis, and write up. BS, JF, SJ, and LY: study design, data analysis, and write up. CG: study design and write up. All authors contributed to the article and approved the submitted version.

## Conflict of Interest

The authors declare that the research was conducted in the absence of any commercial or financial relationships that could be construed as a potential conflict of interest.
